# Political Liberation, Hope, and Social Competition Are the Motor of Secular Trends in Height

**DOI:** 10.1002/ajhb.70095

**Published:** 2025-07-01

**Authors:** Christiane Scheffler, Detlef Groth, Michael Hermanussen

**Affiliations:** ^1^ IBB Human Biology University of Potsdam Potsdam Germany; ^2^ IBB Bioinformatics University of Potsdam Potsdam Germany; ^3^ Pediatrician University of Kiel Kiel Germany

**Keywords:** coherence analysis, community effect on height, competitive growth, hope for a better life, social advancement, strategic growth adjustment

## Abstract

**Background:**

Long‐term improvements in physical living conditions correlate with long‐term trends in height.

**Aim:**

To link temporal characteristics of the secular trend in height with the simultaneous political and economic dynamics.

**Sample and Methods:**

Height of men of the German Armed Forces born between 1865 and 1975 was correlated with indicators of economic prosperity (GDP), nutrition and health (infant mortality), and indicators of social inhomogeneity (income inequality and household wealth share). The time periods before 1916, between 1916 and 1933, 1947, 1973, and after 1989 were separately analyzed. Coherence analysis was used to assess the changes in the temporal trends.

**Results:**

Mean height of young adult men increased by 0.45 mm/year (before 1916), by 2.15 mm/year (1916–1933), by 1.87 mm/year in the early Federal Republic of Germany (FRG) until 1973, by 1.45 mm/year in the late FRG, and by 4 mm/year in East German conscripts after the reunification in 1989. The most substantial height increments occurred in periods of political upheaval and loss of state authority.

**Conclusion:**

The nonlinear pattern of secular height increments in Germany since the late 19th century suggests that political liberation, hope for a better life, and illusions of equity, freedom, justice, and the expectation of social advancement are associated with competitive growth, strategic growth adjustments, and finally, long‐term and substantial secular trends in height.

## 1 Introduction

Plasticity is a common feature in biology. Phenotypic plasticity is defined as the ability of a genotype to express different phenotypes in response to variations in nutritional, environmental and social conditions (West‐Eberhard 2003). Individuals can alter their gene expression, physiology, neurobiology, behavior, and morphology in response to changing conditions. Fine‐grained plasticity in the growth of social animals in response to social positioning within their social networks has been recognized for at least 50 years in many social species and referred to as “strategic growth” or “competitive growth” (Buston and Clutton‐Brock 2022). Members of the same group tend to compete for access to food, sex partners, social position, etc. Competition influences not only interpersonal relationships, but the social network as a whole (Hermanussen et al. 2023). This also seems to apply to humans.

Humans are extremely social and tend to maintain large social networks. Membership in these networks is vital. Perceived group affiliation, group identification, and identity signaling are important features that facilitate in‐group favoritism and out‐group derogation. Affiliations shape common goals and social norms (Tajfel and Turner 1986), and affiliations to social networks have been shown to influence the dynamics of smoking (Christakis and Fowler 2008), the spread of alcohol (Rosenquist et al. 2010), and of obesity (Christakis and Fowler 2007). In recent years, the apparent correlation between individual height and the height of other members of the same social group has attracted particular attention and is referred to as the “community effect on height” (Hermanussen et al. 2014; Hermanussen and Scheffler 2016). “Community effects on height” are defined as the result of social interactions within a group, on growth and body height of its members (Hermanussen and Scheffler 2019) and are particularly obvious in migrants (Bogin et al. 2018; Hermanussen et al. 2025; Scheffler et al. 2021).

**FIGURE 1:**
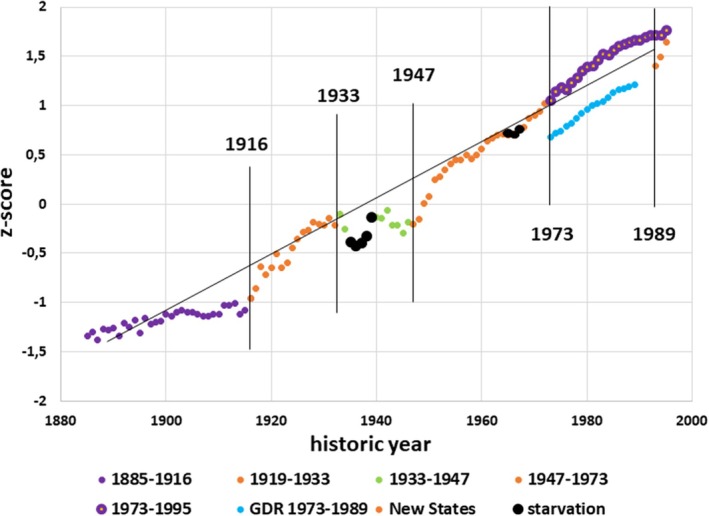
*Z*‐scores for mean height of German men. The *z*‐scores are based on the spectrum of mean heights of all annual cohorts. Colors indicate periods of economic stability (purple), liberation (orange), Nazi dictatorship (green) and GDR (blue). Cohorts born in the periods of starvation during and after World War I and II are indicated by black color. All cohorts are referred to the historic year at age 20, except for the East German cohorts (GDR 1973–1989), which are referred to the year at age 18. The deviation from the linear regression line highlights the nonlinearity of the secular height increments. Vertical bars indicate historical breakpoints.

In this study, we look at “transgenerational” height in the sense of all historically possible body heights that can be achieved within a population that shares a particular gene pool, and consider the spectrum of all possible heights for German men born between 1865 and 1975. For this purpose, we use annual conscript and recruit data, that is, data of young adolescent men; and due to the scarcity of data in the war and post‐war years, also two large cohorts of 19‐year‐old school boys. We name this full, albeit fictitious, spectrum of all historically possible heights, the “transgenerational growth potential.” For German men, this potential has a mean value of 173.0 cm and a standard deviation (SD) of 9.8 cm (Hermanussen et al. 2025) (Figure 1). We are interested in factors that regulate height within this transgenerational spectrum.

Current evidence fails to show that there is any society in history that has ever expressed the full spectrum of its “transgenerational growth potential.” The social community that people share, seems to substantially narrow the potential of achieving a particular height. Individual height is limited within a range close to the average height of his community, that is, community effects appear to protect the individual against being “too tall” or “too short” within its social network (Hermanussen et al. 2025).

Throughout history, body height has changed. Such long‐term height trends tend to be affiliated with similarly long‐term trends in economic prosperity, nutrition, and health, the degree of urbanization (Y. Zhang et al. 2019), the level of education (Arntsen et al. 2023), and the social class (Boyd 1980). These trends have usually been referred to as “secular trends in height.” Secular trends of up to 19 cm (Schönbeck et al. 2013; Van Wieringen 1972), have been observed in the European populations for more than 150 years, and for more than 100 years in many other countries worldwide (NCD Risk Factor Collaboration [NCD‐RisC] 2016).

We are interested in the underlying mechanisms that cause secular trends and studied adult height in German men born between 1865 and 1975. As height is measured at ages of, or greater than 20 years when growth is usually completed and reflects the cumulative effect of all economic, nutritional, social, educational, political, and other factors that are critical to growth, we linked the height of men, born in 1865, to the economic, nutritional, and political situation of the year 1885, we linked height of men, born in 1866, to the year 1886, etc., except for East German cohorts born between 1955 and 1971 in the former German Democratic Republic (GDR). As these cohorts were measured at an age of 17 years, 9 months, we linked their height to the years 1973–1989 (Figure 1).

We then pooled all heights and considered them to reflect the “transgenerational growth potential” of the “German gene pool.” Genetics belongs to the major regulators of growth (Deaton 2007; Yengo et al. 2022), but we consider the “German gene pool” as a stationary factor. This pool has not substantially changed during recent history. Despite the comprehensive territorial reorganization of Germany with permanent territorial losses of around 70 000 km^2^ after 1918 and around 114 000 km^2^ after 1945, and major internal migration from the lost into the remaining territories, the portion of non‐German speaking minorities within this territory had never been more than 10% (Thiel 2012; Wissenschaftlicher Dienste des Deutschen Bundestages 2009). Even before the First World War, when parts of modern Poland, Lithuania, Denmark, and France belonged to the German territory, non‐German‐speaking minorities were comparably small.

Initially, we analyzed the impact of economic situation, diet, and other “hard” physical living conditions that the German population had experienced since the end of the 19th century and which have conventionally been placed in the foreground when discussing the regulation of human growth (Hermanussen et al. 2025). We also considered the general health condition and used infant mortality as a handy substitute. We did not directly address the impact of infectious diseases. Infectious diseases were common in the late 19th century (Leven 1997), but the historic textbooks of pediatrics clearly state that children and adolescents either died from these diseases and never reached adulthood, or survived with no long‐term impairments of health (Gerhardt 1881). Thereafter, we analyzed the impact of “soft” social living conditions on height with particular focus on community effects.

Community effects appear to interfere with the regulation of height in two ways:

1. They should stabilize height as they reduce height differences between people within the same community—adolescents adjust to the height of their peers and parents (Bogin et al. 2018; Hermanussen et al. 2025; Scheffler et al. 2021).
2. They should reflect social dynamics as they respond to an individual's social position among the members of the community—dominant members in mammal societies tend to grow larger than subordinate members (Buston and Clutton‐Brock 2022; Sapolsky and Spencer 1997). Political periods that prescribe fixed class affiliations with little or no opportunities for social advancement across class borders, are expected to show little dynamics in height, whereas periods of political upheaval and loss of state authority are expected to allow for secular changes in height.

Generally speaking, we expect “hope for a better life” (Bogin 2023) as a result of improved social, economic, political and emotional factors (Bogin 2021) and its potential neuroendocrine sequelae that transduce “hope for a better life” into greater skeletal growth (Bogin 2023), to be among the most important regulators of growth (Hermanussen et al. 2022).

As living conditions are closely linked to the political climate, we linked height trends and politics.

### 1.1 The Political Periods

German history since the late 19th century was turbulent. We considered the two World Wars, the beginning of the Nazi dictatorship, the integration of Germany into the common markets of the European countries, and the reunification of Germany as major events in recent German history and defined five breakpoint years.

**1916**

Adult cohorts born before 1896 spent childhood and adolescence in the German Kaiserreich (1871–1918). The Kaiserreich officially ended with the First World War in 1918, but we terminated the imperial period of political and economic stability with the year 1916 when major hardship befell the civilian population. 1916 marks the beginning of the Great Famine (Vincent 1987).

**1933**

Adult cohorts born before 1913 experienced at least parts of their growth in the turbulent period of war and subsequent political liberation (Glatzer and Glatzer 1983), the Great Famine (1916–1919) (Vincent 1987), hyperinflation (1923), and economic failure, accompanied by political radicalization. 1933 marks the beginning of the Nazi dictatorship.

Instead of 1945, we had to choose the year 1947 as breakpoint due to scarcity of conscript data in the war and post‐war years. We used a large cohort of 19‐year‐old school boys from Burg/Fehmarn, North Germany (Träbert 1948) as substitutes. These boys had matured during and shortly after the Second World War.

**1973**

Adult cohorts born in the early Federal Republic of Germany (FRG) after 1953 grew up during the time of the economic miracle (Wirtschaftswunder; Godbersen 2024) into a stabilizing society with new elites, a new lifestyle, and new international connectedness. 1973 marks the end of the Wirtschaftswunder and the oil crisis.

**1989**

The socialist system of East Germany collapsed in 1989, and the GDR joined the FRG 1 year later.

On the basis of these breakpoints, we consider one period of political and economic stability (before 1916). The German Kaiserreich was a feudal society with a three‐class electoral system, little political dynamics, and little or no opportunities for social advancement across social class borders.

Three periods of liberation (1) after the effective collapse of the imperial German economy in 1916 and the beginning of the Great Famine in 1916 to the end of the Weimar Republic (1916–1933); (2) the West German situation after the social and political disintegration after the Second World War to the end of the West German economic miracle (1947–1973); and (3) the East German situation after the German reunification in 1989.

And we considered one ambivalent period of resurgence and consolidation (Streeck 2015) (1973–1995) when, after 1973, new elites were established and the economy stabilized. Yet, these years were still accompanied by ongoing social turbulences in West German society, carried forward by young people from environmental, peace, and anti‐nuclear movements, Third World groups, and women's initiatives who continued gathering to create political alternatives. In 1980, the West German party “The Greens” was founded and entered the Bundestag in 1983. It was not only the full beards and knitted sweaters that were new, but above all, the political style: provocative and relentlessly open and critical—even among themselves (Bündnis 90/Die Grünen 2024).

This period of ambivalence included rapid Europeanization. The social and political development of West German society became intertwined with the respective development of its European neighbors. Economy and mass tourism increasingly connected the people with more than 100 million international arrivals per year after 1970, more than 200 million tourists in 1985, and more than 300 million in 1995 (Areppim AG 2024). The so‐called “Interrail ticket” may serve as a vivid example of the new connectedness. Introduced in 1972, it allowed the young generation up to the age of 21 to travel across Europe for a whole month on a small budget. Following their desire for freedom on the rails, generations of teenagers have since spent their summer vacations in train compartments and station concourses (RND/dpa 2022). All this increasingly blurred the national characteristics after the mid‐1970s.

We excluded the intermediary period of Nazi dictatorship as this period was heterogeneous, with a few years of economic stabilization and political hubris (1933–1939), followed by war, destruction, and foreign occupation.

### 1.2 Living Conditions

In order to describe the general conditions of life in the different historic periods, we chose six indicators for which reasonable amounts of historic data were available (Figure 1).

1. Per capita gross domestic product (GDP) as the key indicator of economic wealth (Rahlf 2022);
2. Meat consumption (Kanerva 2013; Teuteberg 1971) as an indicator of protein supplementation;
3. Milk consumption (Fenton and Owen 1981; IBISworld 2023) as milk has been considered a significant biological stimulator of child (Grenov et al. 2020) and adolescent growth (Wiley 2018);
4. Infant mortality as an indicator of public health (Macrotrends LLC 2024; Mühlichen et al. 2015; Roesle 1925; Statista 2023);
5. Mean number of births per woman as an indicator of family size and an indirect mirror of household economy (Schneider and Dorbritz 2011). As infant mortality and births per woman had decreased in history, we turned the originally negative trends into positive ones for reasons of comparability (reverted data);
6. Percentage of young men and women with university entrance qualification (Müller‐Benedict 2022) as estimates of the general educational level of the population (Impicciatore and Tomatis 2020).

We Added Two “Soft” Indicators of Social Inhomogeneity

7 Gini coefficients for income inequality (Bönke et al. 2015; Gómez León and de Jong 2019). A Gini coefficient of 0 reflects perfect equality, where everyone has the same income, while a Gini coefficient of 1 reflects maximum inequality, where one person gets everything and the others get nothing;
8 Household wealth share of the richest top 1% (Albers et al. 2020; Bartels and Hesse 2024).

In view of the current concepts of growth being affected by economic circumstances, we hypothesize (1) that secular trends in height are linearly related to concurrent economic conditions.

In view of potentially stabilizing social effects on the height of peers, parents, and other persons within the social network who have already achieved their final height (Hermanussen et al. 2025), we hypothesize (2) that sudden political and economic disasters are not immediately reflected in the growth of adolescents.

In view of the potential effects of social dynamics on growth, we hypothesize (3) that political liberation that nurtures illusions of equity, freedom, justice, and the expectation of social advancement stimulates growth, that is, that the prevailing emotional, social and, in the broadest sense, political attitudes of the years in which young adults grew up, is reflected by their height independent of the economic situation.

## 2 Samples and Methods

Height of conscripts and recruits of the German Armed Forces was available for all birth cohorts from 1865 to 1975, except the years 1866–1874, 1877, 1878, 1895–1899, and 1922–1937. Conscript data of the birth cohorts 1865–1922 were obtained from Jaeger et al. (2001), Nowak et al. (2020), and Rass and Rohrkamp (2009). Though the latter data are comparably small in number, with annual cohort sizes ranging between 163 and 832, they were considered representative of the male population recruited between 1936 and 1945 (Rass 2023; Rass and Rohrkamp 2009). Complete height data with conscript numbers per centimeter class were available from the East (birth cohorts 1955–1971; Jäschke, personal communication 1992; sJäschke 1991; Rass 2023; Rass and Rohrkamp 2009) and the West German Armed Forces up to 1994 (birth cohorts 1938–1975; Institut für Wehrmedizinalstatistik und Berichtswesen, Remagen 1995).

East German cohorts were mustered at 17 years 9 months of age with cohort sizes ranging between 112 304 and 143 105 (altogether 2 171 038), West German cohorts and East German cohorts after the German reunification, were mustered at 19 to 20 years, with cohort sizes between 439 046 and 140 688 (altogether 12 458 211). Reichswehr soldiers were mustered not before the age of 20, and in the 19th century, up to the age of 30 years. Except for the East German conscripts before the German reunification, who may not have fully completed growth when drafted, we considered all other men to have reached final adult height.

In the absence of data on conscripts in the years after the Second World War, we substituted data on 19‐year‐old school boys from a 1947 survey from Burg/Fehmarn, North Germany (Träbert 1948) who represented a population of 17 000 city dwellers in an overall more rural community; and data of a 1954 school survey from Munich, Bavaria (Bach 1965) with altogether 190 890 boys of different ages from various social background of which we used the data of more than thousand 19‐year‐old working‐class adults. The 1947 survey represented a mostly rural population that finished growing during World War II though without being directly affected by war‐related events; the 1954 survey represented an urban population that spent its childhood during the war, but as adolescents, also experienced the political and economic turbulences of the immediate postwar period. Missing values were added by flexible imputation (Van Buuren 2019).

### 2.1 Statistics

Living conditions are “nonstationary” time series as they change over time. Nonstationary time series should not immediately be used in regression models as they can create spurious random correlations due to common trends in otherwise unrelated or weakly related variables, and create the illusion of causality, though there is none. A solution to the problem is to convert nonstationary time series into stationary time series. Several techniques have been proposed. Among these are differencing (*Y*′_*t*_ = *Y*_*t*_ − *Y*_*t*_ − 1); logarithmic transformation (*Y*′_*t*_ = ln(*Y*_*t*_)); smoothing by moving averages, detrending using regression models and subtracting the trend from the series *Y*_*t*_, and others. We analyzed annual differences between the variables. We refrained from additional logarithmic transformation, moving averages, and so on, as all these approaches solely focus on the series *Y*_*t*_.

Instead, we used a novel approach that simultaneously combines information from both the *X*‐axis and the *Y*‐axis, called “Coherence analysis” (Mumm et al. 2024). This technique eliminates prevalent linear trends, and visualizes local agreement between serial data within defined time periods. Coherence analysis is based on *z*‐score transformed variables and, similar to Bland–Altman plots (Bland and Altman 2011), relates the difference between two variables to the sum of the variables.

Coherence analysis visualizes serially obtained correlated data. Figures can be simplified in the form of an arrow. The length of the arrow reflects the extent of the *z*‐score changes that the two variables jointly undergo. Arrows that point to the right reflect positive, arrows that point to the left, reflect negative associations. Horizontal arrows denote variables with trends of similar in magnitude. Downward pointing arrows indicate that the *Y*‐variable increases at a higher, upward pointing arrows that the *Y*‐variable increases at a slower rate than the *X*‐variable.

All calculations were performed with Excel, Microsoft Office Standard, and R (R Development Core Team 2024).

## 3 Results

Since the end of 19th century, German men had increased in height by 1.3 mm/year on average, yet with substantial variation in the annual increments that did not reflect the historic dynamics of the living conditions (Figure 1). The height trend was slow during the Kaiserreich with 0.45 mm/year from 165.8 cm (1885) to 167.5 cm (1916); it accelerated thereafter with 2.15 mm/year to 170.5 cm in 1933, but did not further increase during the Nazi dictatorship. Thereafter, height rapidly rose by 1.87 mm/year from 171.0 cm (1947) to 176.8 cm in 1973 and by 1.45 mm/year to 180.1 cm in 1995 (Figure 1). The height of East German conscripts before the reunification of Germany was lower than the height of West German conscripts, partially because they were conscripted at a slightly younger age, but height rose by 4 mm/year for a few years in the New States of Germany after the reunification (Hermanussen 1997). A short negative trend was observed in cohorts born immediately before and during the Great Famine of 1916–1919. These cohorts were up to 1.5 cm shorter than cohorts born immediately prior to or after this period (black dots in Figure 1), though still significantly taller than cohorts born in the Kaiserreich. The slopes of all height trends differ significantly (*p* < 0.001).

**FIGURE 2:**
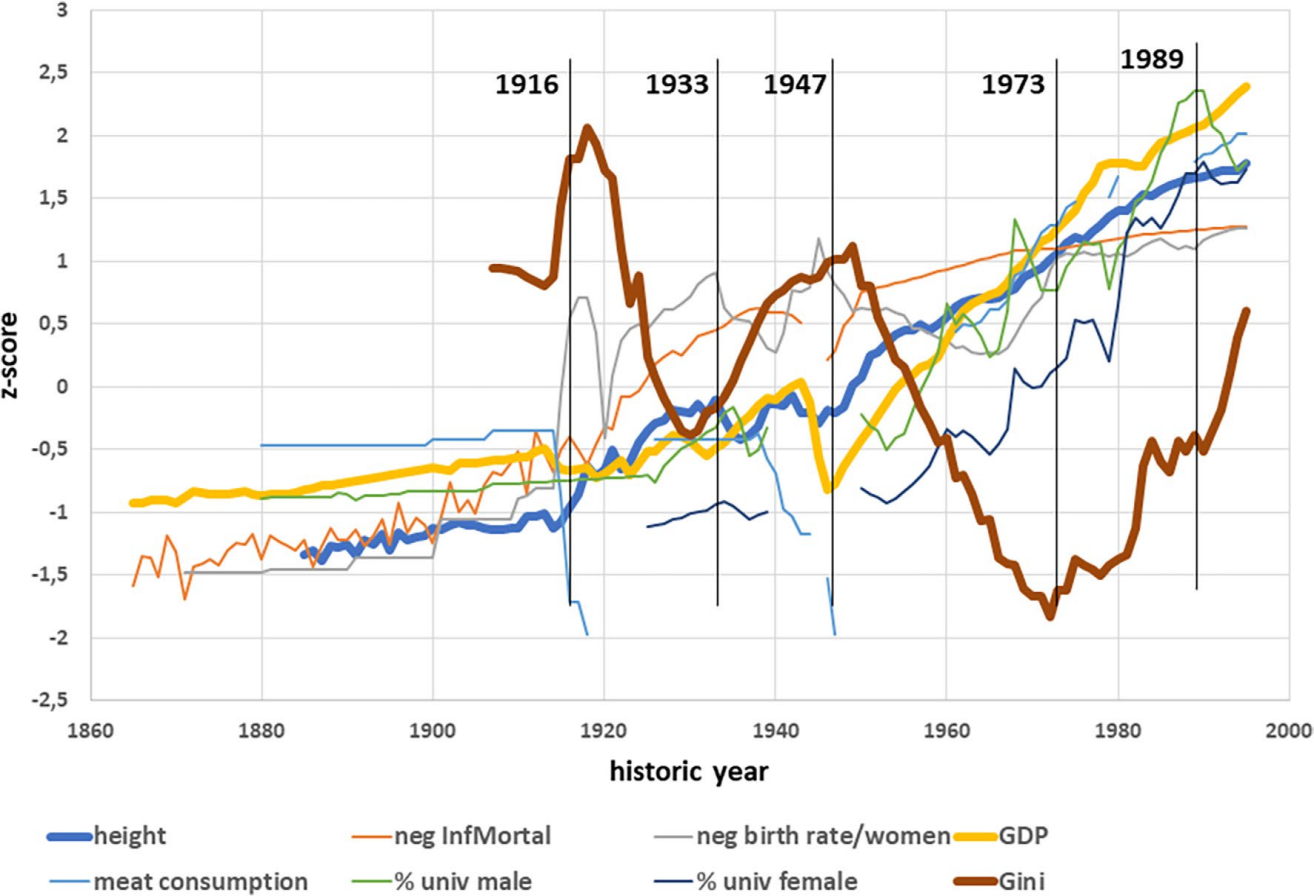
*Z*‐scores for mean adult height, living conditions (GDP, per capita meat consumption), percent male and female students (% univ. male, % univ. female), infant mortality (reverted, neg InfMortal), birth rate per women (reverted, neg birth rate/women), and income inequality (Gini coefficients) between 1885 and 1995. Vertical bars indicate historical breakpoints.

Between 1885 and 1975, living conditions had substantially changed (Figure 2) and were highly interrelated (Table 1). The GDP had increased by a factor of 7; infant mortality had declined 40‐fold, the average number of children per family had dropped from almost 5 to less than 2, the number of male students had exploded by a factor of 15, and the number of female students more than 100‐fold. People eat more meat. Only the consumption of milk showed no discernible long‐term trend. The overall impression appears to suggest that secular trends in height are strongly related to the concurrent economic, nutritional, health, and educational conditions. Yet, this impression is fallacious as explained below.

**TABLE 1:** Correlation coefficients between time (1885–1995), height of German conscripts, infant mortality (InfMortal), birth rate per women, GDP, meat consumption, percent male (% univ. male) and female (% univ. female) university students, and income inequality (Gini).

1885–1995	Time	Height	InfMortal	Birth rate	GDP	Meat	% Univ male	% Univ female
Height	**0.98**							
InfMortal	**−0.97**	**−0.94**						
Birth rate/women	**−0.87**	**−0.83**	**0.91**					
GDP	**0.90**	**0.94**	**−0.80**	**−0.65**				
Meat consumption	**0.77**	**0.84**	**−0.68**	**−0.47**	**0.92**			
% Univ male	**0.92**	**0.94**	**−0.82**	**−0.72**	**0.98**	**0.93**		
% Univ female	**0.92**	**0.94**	**−0.83**	**−0.80**	**0.96**	**0.95**	**0.97**	
Gini	**−0.69**	**−0.72**	**0.76**	**0.41**	**−0.68**	**−0.71**	**−0.63**	−0.29

*Note:* Bold numbers indicate significant correlations (*p* < 0.05). Red colours indicate negative correlations, blue colours indicate positive correlations. The darker, the stronger the association.

The “soft” indicators of social inhomogeneity, Gini index, and household wealth share show a different pattern. Gini coefficients varied around 0.4 with maxima near 0.5 in the Kaiserreich. They rapidly declined in the Weimar Republic, rose again during the Nazi dictatorship, and declined in the early FRG to a minimum of 0.155 in 1972. Meanwhile, income inequality has risen again to levels close to those already seen in the Kaiserreich (Figure 2). The negative correlation between height and the Gini coefficients (*r* = −0.69, *p* < 0.01) indicates toward an overall trend of taller stature in times of less income inequality. Household wealth share of the richest top 1% before the First World War was high with maxima of 48.2% in 1907; it decreased thereafter, but also in the West German democracy, continued to vary at high levels between minima of 20.6% in 1969, and maxima of 25.4% in 1995 indicating that the economic elites in Germany had largely succeeded in maintaining their prosperity over the last century (Albers et al. 2020; Bartels and Hesse 2024).

### 3.1 Stationarity

After converting the nonstationary time series into stationary series of annual differences, most correlations disappeared (Table 2) except for the proportions of male and female students (*r* = 0.66) and the correlations between meat consumption and living conditions. The latter may well be considered as a mirror of the war and post‐war situation. The correlation between height and GDP (*r* = 0.20) became insignificant, suggesting spuriousness in the sense that this association may be due to unobserved confounding factors. The negative correlation between height and the Gini coefficients disappeared (*r* = 0.07, ns).

**TABLE 2:** Correlation coefficients of annual differences between height of German conscripts (1885–1995), infant mortality (InfMortal), birth rate per woman, GDP, meat consumption, percent male (% univ. male) and female (% univ. female) university students, and income inequality (Gini).

Annual diff	Height	InfMortal	Birth rate	GDP	Meat	% Univ male	% Univ female
InfMortal	0.03						
Birth rate/women	−0.05	0.05					
GDP	0.20	−0.03	0.17				
Meat consumption	−0.19	0.14	**0.53**	0.13			
% Univ male	−0.02	0.03	0.05	0.06	0.10		
% Univ female	−0.04	0.08	−0.04	−0.18	**0.44**	**0.66**	
Gini	0.07	0.14	−0.20	−0.12	**−0.46**	0.00	0.05

*Note:* Bold numbers indicate significant correlations (*p* < 0.05). Red colours indicate negative correlations, blue colours indicate positive correlations. The darker, the stronger the association.

### 3.2 *Z*‐Scores and Coherence

**FIGURE 3:**
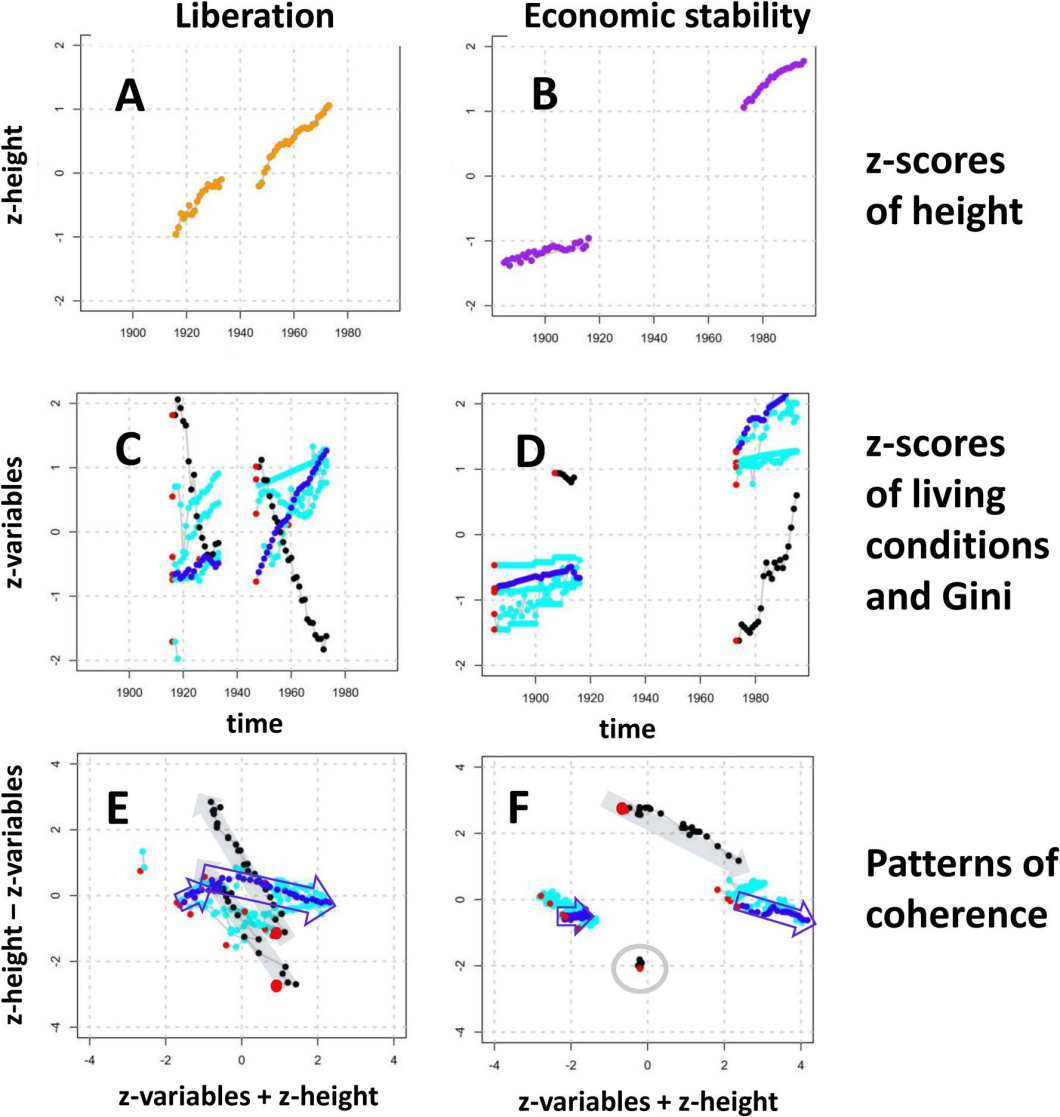
(A and B): *Z*‐scores for mean height in the periods of liberation (orange, born 1896–1913 and 1927–1953), and in the periods of economic stability (purple, born 1865–1896 and 1953–1975). (C and D): *Z*‐scores for indicators of living conditions (dark blue: Key condition GDP; light blue: Meat consumption, infant mortality (reverted), birth rate per woman (reverted), and percent male students). *Z*‐scores for income inequality (Gini coefficients) are given in black. Red points show the first value in each time series to emphasize the temporal direction of the series. For reasons of comparability, height and living conditions are given as “improvements,” that is, *z*‐scores for mean height, GDP, meat consumption, and percent students point into the natural direction (increase with time), while *z*‐scores for infant mortality and births per women are reverted as they decrease with time. (E and F) Coherence plots. Coherence analysis (Mumm et al. 2024) assesses temporal trend changes in serially obtained correlated variables by combining information from both the *X*‐axis and the *Y*‐axis, and visualizes local agreement between serial data within defined time periods. Coherence height*GDP is signalized by blue arrows, coherence height*Gini is signalized by gray arrows. For details, see text.

Z‐score patterns of height and living conditions are associated with social and political background (Figure 3). *Z*‐scores for mean height were almost horizontal in the Kaiserreich (1885–1916, Figure 3B); they rapidly rose in the periods of liberation (1916–1933 and 1947–1973, Figure 3A) and slowed down again in the late FRG (1973–1995, Figure 3B). Z‐scores of the indicators of physical living conditions (blue dots) tended to rise in all periods, though at a very different pace (Figure 3C,D), whereas *Z*‐scores for income inequality (black dots) showed a very different pattern: they substantially dropped in the periods of liberation (Figure 3C) and rose in the periods of economic stability (Figure 3D). Coherence analysis further emphasized the differential associations between height and living conditions.

### 3.3 Coherence During Economically Stable Periods

Compared to later decades, height changed little in the Kaiserreich, so coherence patterns in height*living conditions are generally small, as indicated by the shortness of the horizontal blue arrow (coherence height*GDP), or negligible, as indicated by the gray circle (coherence height*Gini) (Figure 3F). This was different in the late FRG, when both height and living conditions jointly increased. The colored arrows are long, and they point to the right, denoting a positive correlation. The downward direction, however, indicates that both GDP and Gini increase at a higher rate than height; that is, when considering the rapid improvement in living conditions after 1973, the increase in height is comparably small.

### 3.4 Coherence During Periods of Liberation

In the Weimar Republic, height increased rapidly, while physical living conditions improved to a much lesser extent (Figure 3E). The coherence in height*GDP was small and directed upward (short blue arrow). It was only when the economic situation improved in the early FRG that the rate at which GDP increased was greater than the rate at which height increased, as indicated by the long blue arrow pointing to the lower right.

In contrast to the patterns of physical living conditions, the coherences in height*Gini are both directed to the upper left (gray arrows), illustrating the strong negative correlation between Gini and height, and depicting that the significant height improvements in 1916–1933 and in 1947–1973 are accompanied by equally substantial reductions in income inequality. The marked similarity in the coherence in height*Gini in the two periods emphasized the structural resemblance at which the height trends responded to income inequality.

## 4 Discussion

Secular trends in body height have been observed in many countries and are related to economic, political, social, and health‐related factors (Bogin 2021). Bogin and Keep reported on height of over 8000 years of political and economic history in Latin America, and emphasized the impact of living conditions, increased social stratification, and military subjugation by emerging state‐level societies (Bogin and Keep 1999). Skeletal data on European and Near East populations of the last millennium were summarized by Rosenstock et al. (2019). Secular trends in height coincide with living conditions. This is also true for the German society. The mean height of adult German men had increased by 14.3 cm between 1885 and 1995; the GDP had increased by a factor of 7, infant mortality had declined 40‐fold, the average number of children per family had dropped from almost 5 to less than 2, the number of male students had exploded by a factor of 15, the number of female students more than 100‐fold. People eat more meat. Only the consumption of milk showed no discernible long‐term trend. Yet, the primary impression that height depends on the physical conditions of life, is deceptive.

Measurements of height and living conditions ordered by time are time series. As both height and living conditions tend to increase over time, they belong to the category of nonstationary time series and are prone to spurious correlations. The term spurious describes a time series phenomenon that occurs when a series depends on time (Ghouse et al. 2024; Haig 2003). Spuriousness can arise from random or confounding factors and creates the illusion of causality, though there is none. To circumvent this problem, we analyzed the differences between successive annual measurements. This analysis changed the picture: annual differences in economic prosperity, nutrition, and health do not correlate with simultaneous annual changes in mean adult height, indicating the spuriousness of these correlations.

We therefore took a different approach. We defined historic periods that shared certain political and economic characteristics: (1) the German Kaiserreich as a period of political and economic stability; (2) the Weimar Republic, the early years of the FRG, and the first years of the New Federal States (former GDR) after the reunification of Germany in 1989, as three periods of liberation. And we defined the years of the later FRG after the end of the Wirtschaftswunder (economic miracle) and the oil crisis in 1973 as an “ambivalent period” which included the resurgence of former elites and consolidation of economic structures (Streeck 2015) and on the other side, ongoing unrest and persisting provocation by the younger generation and search for political alternatives. We separated these periods by five breakpoint years: 1916, 1933, 1947, 1973, and 1989.

The Kaiserreich before the First World War was a resilient and powerful, feudal structure characterized by orderly political conditions, peace, and a booming industry that accounted for 15% of the global industrial production before the First World War (Scriba 2014). The upper strata of this society were exceptionally prosperous. The richest top 10% owned almost 80% of the national wealth (Albers et al. 2020; Bartels and Hesse 2024) and already in the late 19th century, enjoyed many modern sanitary facilities such as running tub water and wastewater disposal (Lange 2024). In these years, the people were short and height trends did not surpass half a millimeter per year.

This changed around 1916 with the beginning of the Great Famine. In the years of the First World War, the imperial authorities started to tumble and finally collapsed. In the course of the Allied food blockade between 1916 and 1919, food supply was critical (Vincent 1987); Germans lived on 1300 cal per day on average, and more than 750,000 people starved to death. Hyperinflation (1923), economic failure, and the global economic crisis from 1929 prolonged the depression and exacerbated the political and economic problems. Only the health situation had slightly improved: infant mortality and birth rates per woman had declined by some 50% between the beginning of the 20th century and 1933, and the percentage of students had slightly increased, but at 2%–3% was still low compared with modern student numbers.

Despite these conditions, pediatricians described excessive growth in the young generation already in 1916. In 1935, the German pediatrician Koch (1935) summarized: “Size recklessly increases even during marked undernutrition (he refers to the Great Famine 1916–1919) … until the body has wasted its last depot. One might talk about parasitic growth in length” (Hermanussen et al. 2018). Between 1916 and 1933, growth rates in height quadrupled to more than 2 mm per year.

A second major upward trend in height occurred 30 years later in the early FRG. After 1945, when 2.25 million homes were destroyed and 2.5 million damaged, German authorities disbanded, and foreign armies occupied the country, people again suffered from poverty (Baghdady and Würz 2016) and famine (Grossmann 2011), while height began to increase by almost 2 mm/year. The second trend did not start from scratch with 166.8 cm (end of the Kaiserreich), or 168.7 cm (end of the Great Famine in 1919), but with 171 cm, that is, at that height level that the preceding parental cohorts had reached in the 1930s.

The data suggest that adult height does not only reflect the simultaneous or the immediately preceding political and economic situation, but appears to be stabilized by the average height of peers, parents, and other persons who had already achieved their final height. The growth patterns support the second hypothesis. Height trends occur gradually with little variation from year to year. Sudden political and economic disasters are not immediately reflected in the growth of adolescents.

Both periods, the Weimar Republic and the early FRG, emerged after a lost war. They had to deal with the consequences of defeat, with major influx of migrants from the lost territories, and they were democratically organized. The Weimar Constitution of 1919 defined the first German parliamentary democracy. There was universal suffrage for men and women, a Reichstag (parliament), and a president as head of state. In 1949, the German people witnessed the formation of two new countries (1949), the FRG and the GDR. Similar to the Weimar Republic, a federal parliamentary democracy was established in the young FRG, and the economic situation was comparatively critical. The Weimar Republic had to cope with the economic, social, and political consequences of the First World War as well as the harsh conditions of the Treaty of Versailles. The FRG came into being when the country lay in ruins after the Second World War and was confronted with extensive reconstruction, though it soon profited from strong support from the US and the Wirtschaftswunder (Godbersen 2024). Both states suffered from serious political and social tensions. The Weimar Republic was strongly polarized between left‐wing (communist) and right‐wing (nationalist, fascist) forces that destabilized democracy. The young FRG was similarly confronted with political tensions in its early years, including perceived threats from the left‐wing GDR and the East–West conflict, and the strong rejection of democracy by extreme right‐ and left‐wing groups and the incipient RAF (Red Army Fraction) in the 1970s. The main common claim in both the Weimar Republic and the early FRG was the socio‐economic “upgrading” of the individual within a new and democratic country.

A third major upward trend in height with 4 mm per year occurred for a few years among East German conscripts after the reunification (Hermanussen 1997). The reunification was accompanied by major economic and social changes, but not by any similar changes in health and nutritional conditions. In terms of real resources devoted to health services and in terms of health service activities, the former GDR and the pre‐unification FRG seemed to have been almost identical (Hurst 1991). Similarity was also observed in terms of nutrition. Winkler et al. (1997) documented dietary habits in East German men before and after the reunification: mean daily intake of fruit and fruit products increased from 115 to 154 g, intake of bread and baked goods decreased from 286 to 249 g and intake of meat from 91 to 77 g.

The irregular pattern of the secular trend in height since the end of 19th century with significant height improvements during periods of major economic degradation, strongly suggests to reject the first hypothesis. Secular trends in height are not linearly related to concurrent economic conditions. The data rather suggest that the functionality of state authority plays a major role in the regulation of growth. Political liberation, the introduction of democracy, illusions of equity, freedom, justice, and the anticipation of socio‐economic “upgrading” yield competition among the people (T. Zhang and Overbeck 2024). Liberation and social promises yield “hope for a better life” (Bogin 2023).

Liberation, competition, and hope are the key aspects. “Competitive growth” and “strategic growth adjustments” that are frequently observed in social mammals (Buston and Clutton‐Brock 2022) activate the evolutionarily highly conserved neuroendocrine competence for adaptive developmental plasticity (Hermanussen et al. 2022). Yet, liberation, competition, and hope are difficult to measure. Contemporary literature such as the famous description of “the world of yesterday” by Zweig (2016) and newspaper articles can convey impressions that reflect the needs, the spirit, and the social atmosphere of the time these people lived in. But these impressions cannot be scaled. Instead, we used wealth distribution and income inequality as proxies for the social dynamics, for hope and the anticipation of future social improvements. Annual estimates of wealth distribution have been published for recent decades (Albers et al. 2020; Bartels and Hesse 2024), annual estimates of income inequality (Gini index) exist since the end of the German Kaiserreich (Bönke et al. 2015; Gómez León and de Jong 2019).

Income inequality coheres with height. This is particularly evident in the years following political and economic collapse. The social and cultural features known as the “Roaring Twenties” (Gill 1993) were characterized by a general feeling of novelty, modernity, and a break with old traditions. The “Roaring Fifties” (die wilden Fünfziger), termed after the famous novel “Hurra, wir leben noch; Simmel 1981,” describes a similar spirit of optimism following the Second World War, and there are several recent novels about the last years of the GDR, the reunification period, and the hopes and fears of the East German youth (e.g., Tellkamp 2012).

The ambivalent period of resurgence and consolidation (Streeck 2015), accompanied by social turbulence, after the mid‐1970s, was an intermediate state between ongoing unrest pleading for peace and political alternatives and the again widening gap between rich and poor (Blanchet and Martínez‐Toledano 2023). It showed both, political unrest and further progress of average height, and on the other side, the formation of new and very prosperous elites in the FRG. We have arbitrarily chosen the year 1973 as a turning point in the German economy defined by the end of the economic miracle (Wirtschaftswunder; Godbersen 2024), the oil crisis (Bökenkamp 2010), and the accession of Denmark, Ireland, and the United Kingdom to the growing European Community, soon followed by Portugal, Spain, and Greece. The year 1973 denotes the increasing interdependence of the West German economy with the development of its European neighbors and the blurring of national characteristics in favor of growing Europeanization. The increasing flattening of the secular height trend in the later years not only of the FRG, but of several European countries at the end of the 20th century (Hatton and Bray 2010), appears to confirm the link between decreasing growth rates and the fading spirit of optimism in Europe at the end‐20th century (Mak and Garrett 2008). The data support the third hypothesis. Political liberation that nurtures illusions of equity, freedom, justice, and the expectation of social advancement stimulates growth.

Political liberation, upheaval, and loss of state authority are associated with competition and spread hope and prospects of social upgrading, which appear to substantially interfere with the regulation of growth. When competitive growth in periods of political liberation captures the lower social classes, the increase in the growth of these classes begins to intimidate the members of the upper classes and cause “threats of being displaced.” Such threats are strong incentives for above‐normal growth in social mammals (Buston and Clutton‐Brock 2022). The present data suggest that the mutual competition between the classes in political periods of liberation and new “hopes for a better life” (Bogin 2023) led to the excessive gradual secular increase in height that was observed in the Weimar Republic, the early FRG, and the young East German men after the reunification.

The present study challenges the current understanding of the regulation of human growth as it adds community effects to the “various and mutually interrelated environment factors [that] have been proven to influence the secular trend in stature” (Bodzsar and Susanne 1998). Community effects set height targets. They protect against being “too tall” or “too short” (Hermanussen et al. 2025) and explain the blatant absence of tall people in the late German Kaiserreich and the similarly blatant absence of short people in modern European populations. Community effects restrict the magnitude of height increments between the generations and thus, contribute to smoothed patterns of secular height changes even during periods of major changes in the living conditions. Community effects reflect the prevailing emotional, social, and political attitudes and translate “hope for a better life” (Bogin 2023) into body height (Hermanussen et al. 2022). A very popular German song of the mid‐1950s may serve as an example of post‐war hopes for social advancement: “In the waiting room for great happiness, there are many, many people waiting, waiting since yesterday for tomorrow's happiness, and live with wishes for the day after tomorrow (Schwarz 1956).”

### 4.1 Limitations of This Study

We consider the data on conscript height obtained from the Institute of Wehrmedizinalstatistik (Institut für Wehrmedizinalstatistik und Berichtswesen, Remagen 1995) as trustworthy as they are based on more than 90% of all young men conscripted in the FRG between 1957 and 1994. The same applies to the data obtained from Jäschke (Jäschke, personal communication 1992; Jäschke 1991). However, assessing whether the so‐called representative data obtained from the literature are similarly trustworthy is beyond the expertise of the authors. This applies to the height data of young men recruited for the Reichswehr and the Imperial armed forces; and for the indicators of living conditions. There was no reliable information, if during war times, certain privileged strata might have avoided military service. As comparable sources for data on female height are not available, we limited the study to male height.

## 5 Conclusion

Long‐term improvements in living conditions correlate with long‐term trends in height, but these correlations may be spurious. The nonlinear pattern of secular height increments in Germany since the late 19th century reinforces doubts about the prevailing importance of economic prosperity, nutrition, health, and education for growth. The pattern rather suggests that political liberation, hope for a better life, illusions of equity, freedom, justice, and the expectation of social advancement are associated with competitive growth, strategic growth adjustments, and finally, long‐term and substantial secular trends in height.

## Author Contributions

C.S. and M.H. conducted the study, D.G. contributed the statistics, and C.S. and M.H. wrote the manuscript.

## Ethics Statement

The authors have nothing to report.

## Conflicts of Interest

The authors declare no conflicts of interest.

## Data Availability

Data are available by request from authors.
